# Plant-derived extracellular vesicle-like nanoparticles: an emerging immunomodulatory strategy for intervertebral disc degeneration

**DOI:** 10.3389/fimmu.2026.1871902

**Published:** 2026-07-30

**Authors:** Taobo Luo, Moran Suo, Shuang Chen, Zhengbo Wang, Xiangze Huo, Zhihao Xu, Minghan Yu, Wanting Du, Xin Chen, Zhonghai Li

**Affiliations:** 1 Department of Orthopedics, First Affiliated Hospital of Dalian Medical University, Dalian, China; 2 Key Laboratory of Molecular Mechanism for Repair and Remodeling of Orthopedic Diseases, Dalian, China

**Keywords:** immunomodulation, intervertebral disc degeneration, NF-κB signaling, NLRP3 inflammasome, plant-derived extracellular vesicle-like nanoparticles, pyroptosis, sterile inflammation

## Abstract

Intervertebral disc degeneration (IDD), a leading driver of chronic low back pain, has become a major socioeconomic and public health burden due to its high disability rate and substantial treatment costs. Accumulating evidence has redefined IDD as an immune-mediated disorder characterized by the breakdown of immune privilege, infiltration of immune cells, and chronic sterile inflammation driven by innate immune pathways including NF-κB and the NLRP3 inflammasome. Current management strategies, including conservative care and surgery, primarily alleviate symptoms but fail to halt disease progression, while biological therapies are often costly. There is a pressing need for effective, accessible interventions. Plant-derived extracellular vesicle-like nanoparticles (PELNs) present a promising alternative. Rich in bioactive components, PELNs have demonstrated pleiotropic benefits in various diseases by exerting anti-inflammatory, antioxidant, and extracellular matrix (ECM)-enhancing activities, and by inhibiting multiple modes of programmed cell death, including apoptosis, pyroptosis, and ferroptosis. Notably, these nanoparticles act as multi-targeted immunomodulators capable of reshaping the local immune microenvironment—suppressing pro-inflammatory cytokine cascades and dampening inflammasome activation, and shifting macrophage polarization from a pro-inflammatory M1 to a tissue-repair M2 phenotype. These multi-targeted mechanisms align closely with the complex pathophysiology of IDD. Although the direct application of PELNs for IDD remains largely unexplored, their mechanistic profile and inherent advantages position them as a novel and compelling therapeutic candidate to prevent or delay IDD. By targeting the core immunopathological drivers of disc degeneration, PELNs offer a unique opportunity to restore immune homeostasis within this otherwise privileged niche.

## 1 Introduction

Low back pain (LBP) is one of modern society’s most formidable public health challenges. Current World Health Organization estimates indicate that approximately 700 million people worldwide are affected by LBP across varying severity levels (1). Moreover, over 23% of LBP cases progress to chronic intractable conditions. The total annual economic burden of LBP, which includes direct healthcare costs and indirect productivity losses, exceeds hundreds of billions of US dollars worldwide (2). While the etiology of LBP remains incompletely understood and is recognized as multifactorial, intervertebral disc (IVD) degeneration undoubtedly constitutes a significant contributing factor. Epidemiological studies estimate that 26-42% of individuals with degenerative disc disease experience LBP symptoms (3).

IVD, the largest avascular structure in the human body, comprises the nucleus pulposus (NP), annulus fibrosus (AF), and cartilaginous endplate (CEP). Degenerative changes in the structure and function of the IVD are central to the pathogenesis of Intervertebral disc degeneration (IDD). Traditionally regarded as an immune-privileged site due to its avascular nature and physical barriers, the IVD becomes immunologically reactive upon structural failure. Microfractures and delamination expose previously sequestered neoantigens and release damage-associated molecular patterns (DAMPs), triggering innate immune responses that recruit macrophages, neutrophils, and other inflammatory cells into the disc space. This breakdown of immune privilege initiates a self-perpetuating cycle of sterile inflammation, characterized by the activation of the NF-κB pathway and NLRP3 inflammasome, which drives the secretion of key pro-inflammatory cytokines including IL-1β, TNF-α, IL-6, and IL-18. Current clinical management of symptomatic IDD involves conservative approaches, such as analgesics, physical therapy, and anti-inflammatory medications, as well as surgical interventions, all of which are primarily aimed at alleviating neurological symptoms. However, no effective therapy exists to prevent or halt the progression of IVD degeneration. Consequently, the development of targeted, safe, and cost-effective treatments for intervertebral disc degeneration has become a critical imperative.

Plant-derived extracellular vesicle-like nanoparticles (PELNs) are nanoscale particles that contain proteins, lipids, nucleic acids, and small-molecule compounds. They can be isolated from various vegetables, fruits, and medicinal herbs (4–6). Evidence indicates that PELNs modulate cytokine secretion and functionality across diverse immune cells, thereby influencing multiple diseases including cancer, inflammatory bowel disease, and diabetes. Numerous studies have demonstrated that PELNs can modulate pathological mechanisms including inflammation, oxidative stress, apoptosis, pyroptosis, ferroptosis, and dysregulation of extracellular matrix metabolism - all of which are implicated in the development and progression of IDD. Although research on PELNs for IDD treatment remains scarce, their mechanisms demonstrated in animal models of other diseases are highly relevant to its pathological processes. Furthermore, they offer inherent advantages such as biosafety. In particular, the capacity of PELNs to suppress NF-κB signaling, inhibit NLRP3 inflammasome assembly, and modulate immune cell polarization aligns precisely with the immunopathological landscape of the degenerative disc. Consequently, PELN-based therapy represents a promising and transformative strategy for IDD.

## 2 Perspective

**Figure 1:**
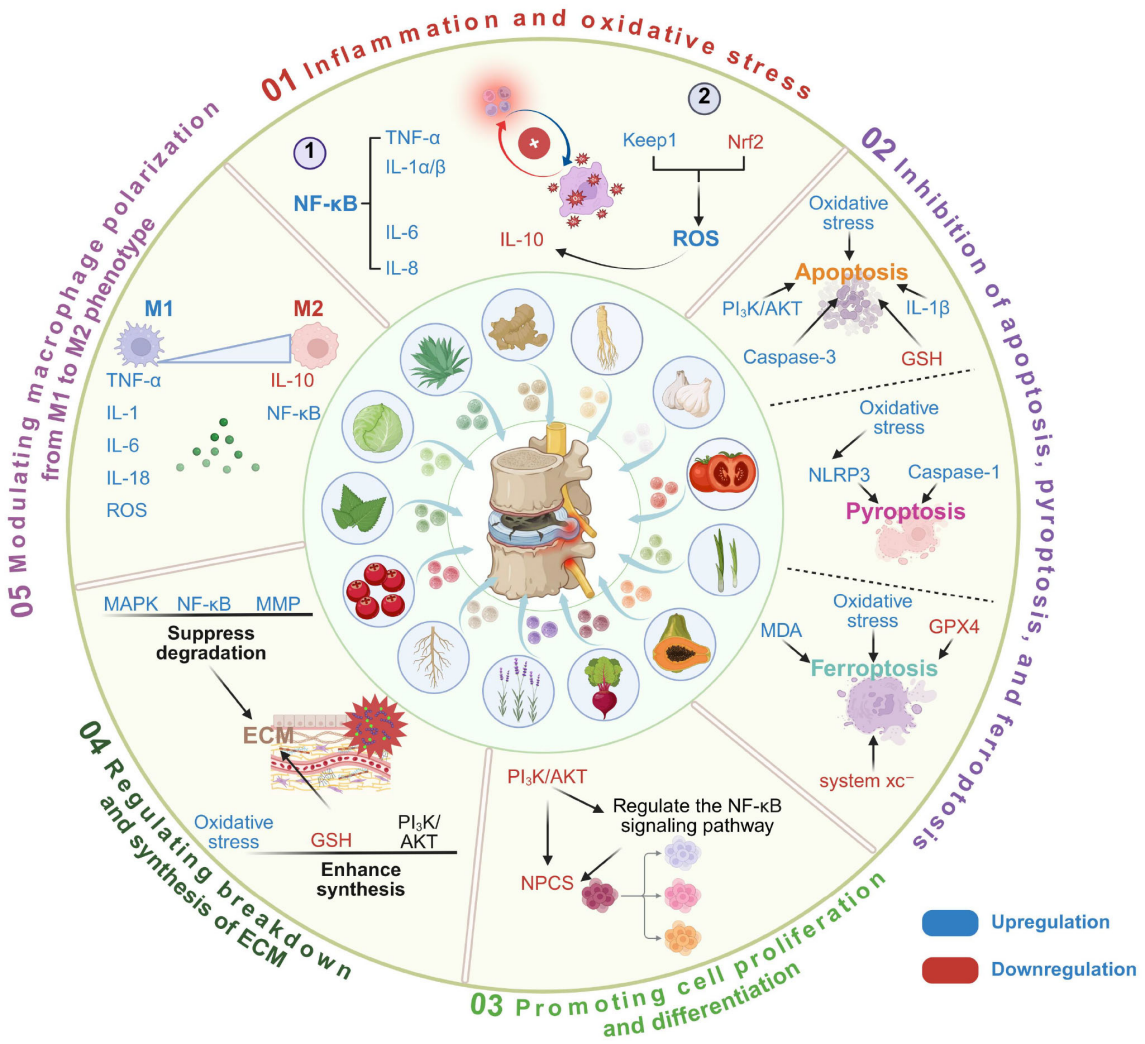
Schematic diagram illustrating the multi-target therapeutic mechanisms of Plant-derived extracellular vesicle-like nanoparticles (PELNs) in the treatment of intervertebral disc degeneration (IDD). The central illustration depicts the degenerative intervertebral disc tissue, with surrounding botanical icons representing diverse sources of plant-derived exosomes. PELNs exert therapeutic effects on IDD through five primary biological mechanisms: (1) regulation of inflammation and oxidative stress, achieved by inhibiting the NF-κB pathway and activating the Nrf2 pathway to suppress pro-inflammatory cytokines and reactive oxygen species (ROS); (2) inhibition of programmed cell death (apoptosis, pyroptosis, and ferroptosis), via regulation of key proteins including caspase-3, NLRP3, and GPX4, as well as enhancement of antioxidant systems such as GSH; (3) promotion of nucleus pulposus cell proliferation and differentiation, mediated through the activation of the PI3K/AKT signaling pathway and modulation of NF-κB activity; (4) maintenance of extracellular matrix (ECM) homeostasis, by suppressing ECM degradation (e.g., via inhibition of MMPs) and enhancing ECM synthesis through anti-oxidative stress mechanisms; (5) modulation of macrophage polarization from the pro-inflammatory M1 phenotype to the anti-inflammatory M2 phenotype, thereby reducing local inflammatory responses. Blue and red symbols indicate upregulation and downregulation of molecular targets or signaling pathways, respectively.

IDD is driven by a cascade of interconnected pathological processes, notably chronic inflammation, oxidative stress, diverse modes of programmed cell death, and extracellular matrix (ECM) breakdown. From an immunological standpoint, the degenerative cascade is initiated and perpetuated by the loss of immune privilege and the subsequent activation of innate immune sensors. DAMPs released from degraded ECM and stressed cells engage pattern recognition receptors such as TLRs, fueling the NF-κB and inflammasome signaling axes that lie at the heart of disc inflammation. An optimal treatment must not only be safe but also multi-targeted, addressing these concurrent mechanisms without compromising spinal function. Existing therapies are inadequate as they lack this comprehensive scope. In contrast, PELNs exhibit pleiotropic effects, such as anti-inflammatory, antioxidant, and anti-apoptotic activities, that directly counter IDD pathogenesis. Moreover, their immunomodulatory properties extend to the regulation of immune cell infiltration and the resolution of sterile inflammation, which are critical for interrupting the chronic inflammatory state of the degenerating disc. These attributes collectively establish PELNs as a compelling novel therapeutic strategy (**Figure 1**). Therefore, we hypothesize that PELNs can attenuate the progression of intervertebral disc degeneration.

## 3 Therapeutic potential of PELNs in IDD

The therapeutic promise of PELNs in IDD stems from their capacity to intervene at multiple nodes of the immune-inflammatory cascade. Below we detail how these nanoparticles target key immunopathological processes implicated in disc degeneration.

### 3.1 Targeting inflammation and oxidative stress

The pathogenesis of IDD is propelled by a self-amplifying vicious cycle between inflammation and oxidative stress. This process is orchestrated by a well-characterized network of pro-inflammatory cytokines, including tumor necrosis factor-α (TNF-α), interleukin-1α/β (IL-1α/β), IL-6, and IL-8. Notably, TNF-α and IL-1β potently stimulate the production of IL-6 and IL-8 within IVD cells, establishing a positive feedback loop that sustains a localized inflammatory microenvironment (7).Concurrently, inflammation-induced mitochondrial dysfunction in IVD cells serves as a primary source of reactive oxygen species (ROS). The resulting ROS accumulation further promotes inflammatory mediators, thereby reinforcing a pathogenic cycle that ultimately accelerates IDD progression (8, 9).

These findings suggest that PELNs may have the potential to disrupt this vicious cycle by targeting inflammatory and oxidative stress pathways, although direct evidence in IVD cells or IDD models is currently lacking. Importantly, the mechanistic evidence summarized below has been derived predominantly from models of colitis, dermal inflammation, and other highly vascularized and immunocompetent tissues. Direct validation in the avascular, hypoxic, hyperosmotic, and mechanically stressed IVD microenvironment is currently lacking. Therefore, the findings discussed here serve as a conceptual framework and hypothesis-generating rationale, rather than direct evidence of efficacy in intervertebral disc degeneration.

Empirical evidence indicates that PELNs from diverse botanical sources exert potent anti-inflammatory effects. For instance, PELNs derived from fresh Rehmanniae Radix significantly reduce the production of pro-inflammatory cytokines such as IL-1β, IL-6, and TNF-α (10). Similarly, lavender-derived PELNs effectively lower levels of IL-1β, IL-6, and TNF-α (11), while papaya-derived PELNs downregulate mRNA expression of IL-1B and IL-6 and upregulate that of the anti-inflammatory cytokine IL-10 (12). The chitosan-polyvinyl alcohol nanomembrane incorporating nanovesicles from Aloe barbadensis, Azadirachta indica, and Zingiber officinale (OXY-NM^Aloe^) also demonstrates a significant reduction in IL-6 and TNF-α (13). Besides, the nuclear factor kappa B (NF-κB) signaling pathway functions as a master regulator of inflammatory responses in IDD (14), and multiple PELNs exhibit their anti-inflammatory effects through its modulation. Ginger-derived exosome-like nanoparticles (G-ELNs) suppress inflammation by downregulating NF-κB, IL-6, IL-8, and TNF-α expression (15–17), and ginseng-derived nanoparticles (GENs) inhibit TNF-α and IL-6 via NF-κB blockade (18).Notably, garlic exosome-like nanovesicles (GaELNVs) not only reduce IL-6, IL-1β, and TNF-α levels but also inhibit NF-κB activation and substantially decrease the secretion of the pro-inflammatory cytokine IL-18 (19).

Complementing their anti-inflammatory properties, PELNs also address oxidative stress through multiple antioxidant pathways. A key mechanism involves the activation of the nuclear factor erythroid 2-related factor 2 (Nrf2) axis, whose reduced expression during IDD correlates with elevated ROS levels. Therapeutic Nrf2 activation effectively mitigates oxidative stress in nucleus pulposus cells (NPCs) (20). For instance, coriander-derived nanovesicles activate Nrf2 signaling to enhance antioxidant enzymes (21), while aloe-derived nanoparticles (ADNPs) promote Nrf2 nuclear translocation to alleviate oxidative stress (22). Furthermore, tomato-derived miR164a/b-5p suppresses Keap1 mRNA, thereby amplifying Nrf2-mediated responses (23), and G-ELNs bind specifically to multiple oxidative stress-related target mRNAs (24). Beyond Nrf2 activation, mitochondrial protection is achieved by Perilla frutescens-derived PELNs that directly reduce ROS levels (25). The therapeutic relevance of these antioxidant mechanisms is strongly supported by evidence that mulberry leaf-derived PELNs alleviate oxidative damage in an established injury model (26), highlighting their potential to counteract oxidative stress in IDD. By mitigating the inflammatory and oxidative microenvironment, PELNs may indirectly protect NPCs from cytokine-induced catabolism and senescence, thereby preserving the functional cell population within the disc.

### 3.2 Inhibiting multiple modes of programmed cell death

Indirect evidence suggests that PELNs may have potential protective effects against multiple modes of programmed cell death relevant to IDD, including apoptosis, pyroptosis, and ferroptosis. As with the anti-inflammatory mechanisms, nearly all experimental evidence on PELNs-mediated cytoprotection has been obtained from non-IDD models. It remains to be investigated whether these protective effects can be reproduced in NPCs under the unique conditions of high osmotic pressure, acidic pH, and hypoxia in degenerated intervertebral discs. The following discussion should be regarded as the pharmacological basis for future specific studies targeting IDD. Given that excessive programmed cell death is a primary cause of NPC depletion during IDD, these anti-apoptotic, anti-pyroptotic, and anti-ferroptotic properties of PELNs could directly contribute to maintaining NPC survival within the degenerative niche.

#### 3.2.1 Inhibiting apoptosis

ROS accumulation triggers oxidative stress accompanied by mitochondrial dysfunction and diminished Nrf2 signaling, ultimately inducing apoptosis through the mitochondrial pathway (27). In IDD, activation of the PI3K/Akt signaling pathway effectively attenuates oxidative stress-induced apoptosis in NPCs (28). Furthermore, modulating the MAPK/NF-κB signaling axis suppresses IL-1β-induced inflammation, a key driver of apoptosis, thereby ameliorating apoptosis in NP cells within a disc degeneration context (29).

Recent advances in PELNs reveal their potent anti-apoptotic properties. Specifically, PELNs derived from cabbage (Cabex) and red cabbage (Rabex) significantly inhibit inflammatory responses by reducing pro-inflammatory cytokines IL-6 and TNF-α, thereby suppressing caspase-3 activation and subsequent apoptosis induction (4). Similarly, cranberry-derived PELNs (Va-exos) exhibit multifaceted protective effects by markedly reducing apoptosis and reactive oxygen species accumulation in murine cells (5). Mechanistic studies demonstrate that hybrid balloon flower root-derived PELNs with liposome-loaded silymarin achieve pharmacological inhibition of the MAPK signaling cascade coupled with effective suppression of apoptotic pathways (30). G-ELNs modulate the PI3K/AKT-2 signaling pathway through their miRNA components, thereby inhibiting cellular apoptosis (24). These findings collectively suggest that PELNs may possess anti-apoptotic properties through oxidative stress mitigation and inflammatory response control. These properties suggest that PELNs could contribute to delaying IDD progression, though dedicated studies using NPCs and animal IDD models are required to confirm this possibility.

Additionally, glutathione (GSH), as the primary antioxidant maintaining cellular redox homeostasis, has been demonstrated to suppress H2O2-induced apoptosis in human NPCs (31). The OXY-NM^Aloe^ increases in GSH levels (13). Beta vulgaris-derived exosome-like nanovesicles significantly increase both the GSH/oxidized glutathione (GSSG) ratio (32).

#### 3.2.2 Inhibiting cell pyroptosis

Pyroptosis, a form of programmed cell death, can be initiated by oxidative stress through ROS generation and activation of oxidative stress-associated signaling cascades (33). A pivotal mechanism of oxidative stress-induced pyroptosis involves NLRP3 inflammasome activation. Therapeutic suppression of either oxidative stress or the NLRP3 inflammasome effectively inhibits pyroptotic cell death, thereby mitigating IDD progression. Treatment with GaELNVs resulted in a significant reduction in NLRP3 activation and Caspase-1 maturation (19). Perilla frutescens-derived PELNs and Va-exos demonstrated mitigating ROS levels (5, 25). PELNs derived from Houttuynia cordata effectively attenuate pyroptotic cell death via targeted modulation of the NLRP3 inflammasome complex within the NOD-like receptor signaling cascade (34).

The canonical pyroptosis pathway initiates with caspase-1 activation, leading to gasdermin-mediated pore formation in the plasma membrane. Subsequent osmotic lysis releases bioactive IL-1β and IL-18, amplifying inflammation (35). G-ELNs and fresh Rehmanniae Radix effectively reduce IL-1β levels (10, 24). GaELNVs not only significantly inhibit NLRP3 inflammasome activation and Caspase-1 maturation, but also reduce IL-6, IL-1β, TNF-α levels and inhibit NF-κB, while substantially decreasing the secretion of pro-inflammatory cytokine IL-18 (19). If validated in disc cells under degeneration-mimicking conditions, these anti-pyroptotic effects could mitigate IDD progression.

#### 3.2.3 Inhibiting cell ferroptosis

Ferroptosis, a regulated cell death pathway characterized by iron-dependent lipid peroxidation, has emerged as a critical factor in IDD pathogenesis, significantly driven by oxidative stress (36–38). Accordingly, PELNs that bolster cellular antioxidant defenses show promise in mitigating this process. Shallot and garlic-derived exosomes activate the Nrf2 pathway (39), while ADNPs promote Nrf2 nuclear translocation through the Nrf2/ARE pathway, alleviating oxidative stress. Similarly, miRNA164a/b-5p within tomato-derived exosome-like nanovesicles reduces Keap1 mRNA expression, enhancing Nrf2-mediated antioxidant gene expression (23). Beyond Nrf2 activation, certain PELNs directly target the executioners of ferroptosis. Beta vulgaris-derived exosome-like nanovesicles significantly decrease malondialdehyde (MDA) levels while increasing the GSH/GSSG ratio and the expression of system xc- and glutathione peroxidase 4 (GPX4), effectively reversing doxorubicin-induced ferroptosis (32). Green onion-derived PELNs inhibit glutamate-induced lipid peroxidation, reduce intracellular iron accumulation, and upregulate GPX4, thereby preventing ferroptotic cell death (40). The reduction of MDA, a key lipid peroxidation product, is also observed after treatment with OXY-NM^Aloe^ and ADNPs (13, 22). While these anti-ferroptotic effects are promising, none of these studies were performed in IDD models, and the relevance of these mechanisms to ferroptosis in the unique disc microenvironment remains to be established. Nevertheless, these findings provide a pharmacological rationale for investigating PELNs in IDD-related ferroptosis.

### 3.3 Promoting cell proliferation and differentiation

Beyond mitigating cell death, restoring the cellularity of the intervertebral disc through the promotion of proliferation and differentiation is a crucial therapeutic goal for IDD. Evidence from non-IDD models indicates that certain PELNs can stimulate regenerative processes. For instance, exosomes from Cissus quadrangularis effectively induce the proliferation and differentiation of human mesenchymal stem cells (41), while PELNs derived from cabbage (Cabex) and red cabbage (Rabex) directly promote cellular proliferation (4). The regenerative potential of PELNs may be further amplified through the modulation of key signaling pathways implicated in IDD. Specifically, they may engage the PI3K/Akt pathway, whose activation is known to ameliorate impaired NPCs proliferation under oxidative stress (42), and influence the multifunctional transcription factor NF-κB, which also modulates cell proliferation (43). Through these pro-proliferative and pro-differentiation effects, PELNs could directly replenish the NPC population and restore the cellularity required for disc homeostasis.

### 3.4 Regulating breakdown and synthesis of ECM

A cornerstone of IDD pathology is the dysregulation of ECM metabolism, characterized by excessive degradation and inadequate synthesis or repair. PELNs may address this by simultaneously inhibiting catabolic processes and promoting anabolic and protective mechanisms. To curb excessive degradation, PELNs target the upstream drivers. They suppress pro-inflammatory cytokines through specific PELNs, such as GENs and GaELNVs (18, 19), as well as the oxidative stress that activates MAPK/NF-κB signaling and MMP production (19, 44, 45). This direct inhibition is exemplified by OXY-NM^Aloe^, which reduces excessive degradation by 40% (13). To enhance ECM preservation and synthesis, PELNs employ complementary strategies. They boost antioxidant defenses, for example, by increasing GSH with specific PELNs such as OXY-NM^Aloe^ and BELNVs (13, 32), and activate crucial pro-survival pathways. A key example is the PI3K/Akt pathway, which is modulated by G-ELNs to enhance NP cell viability and directly inhibit factors that impair ECM secretion (24, 28, 42). By preserving ECM integrity, PELNs not only maintain the structural scaffold of the disc but also restore the native matrix signals that are essential for NPC survival and function.

### 3.5 Modulating macrophage polarization from M1 to M2 phenotype

The breakdown of immune privilege in the degenerating IVD is critically driven by the infiltration and polarization of macrophages (46). M1 macrophages mount a pro-inflammatory response by secreting TNF-α, IL-1, IL-6, IL-18, and ROS, whereas M2 macrophages shift toward anti-inflammatory and tissue-repair functions by secreting IL-10 and TGF-β, the principal anti-inflammatory factors regulating inflammation in IDD. Shifting the M1/M2 balance toward an M2-dominant phenotype therefore represents a promising immunomodulatory strategy for IDD (47).

Notably, the capacity of PELNs to suppress NF-κB signaling (48) and upregulate IL-10 offers a direct mechanistic basis for promoting M1-to-M2 repolarization. NF-κB activation is a master driver of M1 polarization, and its inhibition has been shown to favor the M2 phenotype (49). Consistently, multiple PELNs, including garlic-derived GaELNVs, ginger-derived G-ELNs, and ginseng-derived GENs, potently inhibit NF-κB activation (15, 17–19) and reduce downstream M1-associated cytokines, including TNF-α, IL-1, IL-6, IL-18, and ROS (10–13, 15–19), thereby suppressing M1 polarization. Simultaneously, certain PELNs such as papaya-derived PELNs upregulate the anti-inflammatory cytokine IL-10 (12), a key inducer of M2 polarization and a hallmark of M2-mediated tissue repair. By concurrently suppressing the NF-κB-driven M1 program and promoting IL-10-mediated M2 functions, PELNs could effectively reshape the macrophage landscape within the degenerative disc, breaking the self-perpetuating cycle of sterile inflammation and restoring a pro-repair immune microenvironment. It should be acknowledged that direct evidence of PELN-mediated macrophage polarization within the avascular and harsh IVD microenvironment is currently lacking, and dedicated IDD-specific studies are required to validate this mechanism. Through this M2-skewing effect, PELNs could establish an NPC-supportive paracrine environment in which IL-10 and TGF-β from M2 macrophages promote NPC survival, suppress MMP-mediated matrix degradation, and foster tissue repair.

## 4 Challenges and future perspectives

While PELNs hold significant therapeutic potential, their clinical translation for IDD faces several formidable challenges. A particularly critical issue is the “delivery paradox” inherent to the avascular IVD. Systemic administration (oral or intravenous) is unlikely to achieve therapeutic concentrations of PELNs within the disc, as the absence of a vascular supply and the dense annulus fibrosus severely limit passive diffusion and convective transport. Conversely, direct intradiscal injection, the most straightforward route to local delivery, requires needle puncture through the annulus fibrosus, which is itself a well-established inducer of disc degeneration in animal models, creating a clinical dilemma in which the delivery method may exacerbate the pathology it aims to treat.

Two potential strategies may help circumvent this paradox. First, minimally invasive delivery platforms employing ultrafine-gauge needles or hollow microneedle arrays can substantially reduce the size of the annular defect compared with conventional injection needles. These can be paired with *in situ* gelling hydrogels that rapidly solidify to seal the puncture tract. For example, thermosensitive chitosan/β-glycerophosphate systems or photo-crosslinkable gelatin methacryloyl (GelMA) can be injected as a low-viscosity liquid and cured *in situ*, achieving both PELN delivery and immediate sealing of the annular puncture site. Second, degenerating discs often harbor pre-existing structural defects, including annular fissures and endplate microfractures. These naturally occurring openings could be exploited as direct delivery routes for PELN formulations, thereby avoiding iatrogenic puncture altogether. Both strategies remain entirely unexplored for PELNs in IDD and represent critical avenues for future investigation. To further enhance clinical translatability, these delivery procedures could be performed under fluoroscopic or ultrasound guidance to ensure accurate placement, and the compatibility of PELNs with radiopaque contrast agents should be verified.

In addition to delivery barriers, the unique physicochemical microenvironment of the degenerating disc, including low pH, elevated osmolarity, severe hypoxia, and dynamic mechanical loading, may profoundly affect PELN stability, cellular uptake, and cargo bioactivity, and these microenvironment-specific factors must be systematically evaluated before clinical translation can be considered. Dynamic mechanical loading during spinal motion, for instance, could physically disrupt PELNs or alter the release kinetics of hydrogel-encapsulated nanoparticles, representing an additional challenge that has not yet been addressed. Similarly, the acidic pH of degenerated discs may alter PELN surface charge and colloidal stability, while elevated osmolarity could induce vesicle shrinkage and cargo leakage; how these physicochemical stresses collectively affect PELN cellular uptake and intracellular trafficking remains to be investigated. Although direct evidence in the IVD context remains limited, several findings suggest that PELNs possess inherent structural resilience against these harsh conditions. Ginger-derived PELNs, for instance, can withstand the strong acidic and enzymatic environment of the gastrointestinal tract, indicating potential structural stability under the low pH conditions of degenerating discs (50). The hypertonic environment may cause PELN shrinkage and increase membrane permeability, leading to leakage of internal components; however, long-chain fatty acids and sterol esters within PELN lipid bilayers may strengthen membrane structure and partially resist such deformation. PELNs can also attenuate hypoxia-induced ferroptosis by regulating HIF-1α and HIF-2α-mediated lipid peroxidation (51).

Encouragingly, composite hydrogels engineered with microenvironment-responsive properties offer a complementary solution to these challenges. ROS-responsive hydrogels incorporating thioketal or boronic ester linkages can degrade in the oxidative disc environment to achieve on-demand PELN release. pH-sensitive hydrogels based on Schiff base or acetal bonds can respond to the acidic conditions of the degenerating disc to trigger controlled cargo liberation. To counteract hyperosmotic stress, osmoprotectant-loaded hydrogels incorporating betaine or trehalose could stabilize both the hydrogel network and the encapsulated PELNs, preventing vesicle shrinkage and cargo leakage (52). For the severely hypoxic environment, oxygen-generating hydrogels containing catalase or manganese dioxide nanoparticles could alleviate local hypoxia (53), thereby sustaining PELN bioactivity and preserving NPC endocytic function. These microenvironment-adaptable composite hydrogel systems have been investigated in other contexts but remain entirely unexplored for PELN-based IDD therapy, representing an exciting frontier for future translational research.

Beyond delivery and microenvironmental challenges, the bioactivity and stability of these plant-derived molecules in humans require thorough validation. This is crucial because interspecies differences may limit extrapolation from animal models. The immunomodulatory effects of PELNs are context-dependent and may vary based on the specific immune status of the host. In the unique immune microenvironment of the degenerating disc, characterized by immune cell infiltration, the precise immunological outcomes of PELN administration warrant careful characterization to avoid unintended immune activation. Furthermore, the inherent heterogeneity and lack of standardization in PELNs pose a significant bottleneck. These issues result from variations in plant species, growth conditions, and extraction methods, leading to batch-to-batch differences in particle size distribution, cargo composition, and bioactivity. Therefore, rigorous quality control metrics, such as nanoparticle tracking analysis (NTA) for particle concentration and size, and functional potency assays, must be established. Reference standards analogous to those proposed for mammalian extracellular vesicles (e.g., MISEV guidelines) may serve as a useful template, complemented by plant-specific parameters such as botanical authentication and proteomic/lipidomic profiling.

Finally, the foundational parameters of dosage and long-term safety remain unestablished, despite assumptions of safety due to their natural origin. Comprehensive dose-response studies using IDD-relevant models, local pharmacokinetic profiling with fluorescently labeled PELNs, and long-term immunotoxicity assessments, including evaluation of potential hypersensitivity reactions and effects on systemic immune homeostasis, are essential prerequisites for clinical development (54).

## 5 Conclusion

In summary, the pathogenesis of IDD is defined by a self-perpetuating cycle of inflammation and oxidative stress, mediated through dysregulated signaling pathways that collectively lead to cell death and matrix destruction. Central to this destructive cycle is the breakdown of immune privilege, which transforms the disc from a quiescent, avascular structure into an arena of active innate immune engagement. The interplay between DAMPs, TLR signaling, NF-κB activation, and inflammasome-driven cytokine release constitutes the immunopathological backbone of disc degeneration, and the infiltration and polarization of macrophages further amplify this sterile inflammatory cascade. No clinically available therapies currently exist that effectively halt the progression of intervertebral disc degeneration while fully preserving spinal function. In this context, PELNs emerge as a compelling therapeutic candidate. Their pleiotropic nature enables them to simultaneously target the very pathways that drive IDD, including suppressing NF-κB-driven inflammation, inhibiting NLRP3-mediated pyroptosis, blocking ferroptosis, protecting ECM integrity, and shifting macrophage polarization from a pro-inflammatory M1 to a tissue-repair M2 phenotype. This synergistic multi-target approach offers advantages that single-target therapies cannot match. Crucially, PELNs modulate the immune landscape of the degenerative niche—dampening inflammatory signaling, inhibiting immune-mediated cell death, and potentially restoring the delicate balance between tissue repair and immune surveillance. Although significant delivery and microenvironmental challenges remain to be addressed, the convergence of PELN biology with advanced hydrogel-based delivery systems represents an exciting frontier for future research. When combined with their inherent biosafety, the case for PELNs is compelling. However, it must be emphasized that direct experimental validation of PELN efficacy in IDD-specific models remains a prerequisite before clinical translation can be considered. We therefore propose that PELN-based therapy represents a novel and highly promising strategic direction worthy of future investigation.

## Data Availability

The raw data supporting the conclusions of this article will be made available by the authors, without undue reservation.
